# Planning and Optimization of Software-Defined and Virtualized IoT Gateway Deployment for Smart Campuses

**DOI:** 10.3390/s22134710

**Published:** 2022-06-22

**Authors:** Divino Ferreira, João Lucas Oliveira, Carlos Santos, Tércio Filho, Maria Ribeiro, Leandro Alexandre Freitas, Waldir Moreira, Antonio Oliveira-Jr

**Affiliations:** 1 Campus Senador Canedo, Federal Institute of Education, Science and Technology of Goiás (IFG), Senador Canedo 75250-000, Brazil; divino.alves@ifg.edu.br; 2 Institute of Informatics (INF), Federal University of Goiás (UFG), Goiânia 74690-900, Brazil; joaooliveira@discente.ufg.br; 3 Campus Palmas, Federal Institute of Education, Science and Technology of Tocantins (IFTO), Palmas 77021-090, Brazil; carlosedu@ifto.edu.br; 4 Institute of Biotechnology (IBiotec), Federal University of Catalão (UFCAT), Catalão 75705-220, Brazil; tercioas@ufg.br; 5 Institute for Systems and Computer Engineering, Technology and Science (INESC-TEC), 4200-465 Porto, Portugal; maria.r.ribeiro@inesctec.pt; 6 Campus Inhumas, Federal Institute of Education, Science and Technology of Goiás (IFG), Inhumas 75402-556, Brazil; leandro.freitas@ifg.edu.br; 7 Fraunhofer Portugal AICOS, 4200-135 Porto, Portugal; waldir.junior@fraunhofer.pt

**Keywords:** Internet of Things (IoT), smart campus, IoT gateway, cluster, optimization

## Abstract

The Internet of Things (IoT) is based on objects or “things” that have the ability to communicate and transfer data. Due to the large number of connected objects and devices, there has been a rapid growth in the amount of data that are transferred over the Internet. To support this increase, the heterogeneity of devices and their geographical distributions, there is a need for IoT gateways that can cope with this demand. The SOFTWAY4IoT project, which was funded by the National Education and Research Network (RNP), has developed a software-defined and virtualized IoT gateway that supports multiple wireless communication technologies and fog/cloud environment integration. In this work, we propose a planning method that uses optimization models for the deployment of IoT gateways in smart campuses. The presented models aimed to quantify the minimum number of IoT gateways that is necessary to cover the desired area and their positions and to distribute IoT devices to the respective gateways. For this purpose, the communication technology range and the data link consumption were defined as the parameters for the optimization models. Three models are presented, which use LoRa, Wi-Fi, and BLE communication technologies. The gateway deployment problem was solved in two steps: first, the gateways were quantified using a linear programming model; second, the gateway positions and the distribution of IoT devices were calculated using the classical K-means clustering algorithm and the metaheuristic particle swarm optimization. Case studies and experiments were conducted at the Samambaia Campus of the Federal University of Goiás as an example. Finally, an analysis of the three models was performed, using metrics such as the silhouette coefficient. Non-parametric hypothesis tests were also applied to the performed experiments to verify that the proposed models did not produce results using the same population.

## 1. Introduction

Information technology has become essential in the daily lives of people and businesses and the Internet of Things (IoT) concept directly contributes to changes in everyday life [1,2,3,4,5,6,7]. The IoT is a communication paradigm in which objects communicate with each other and with users via network communication technologies, mostly wireless networks [8]. This paradigm enables interaction between several devices (called things), such as electronic appliances, home appliances, vehicles, hospital equipment, sensors, surveillance cameras, etc. The technologies that are used in the IoT context directly contribute to the evolution of services that are associated with smart cities and smart campuses. Hence, smart campuses are integrated work, study, and living environments that are based on the Internet of Things [9]. A multitude of data types that correspond to distinct applications are transmitted over the IoT infrastructure and need to be processed in different units [10].

Two of the main challenges that are analyzed and faced by IoT are interoperability and heterogeneity [11]. One solution to these challenges is the deployment of IoT gateways that support multiple communication technologies [12]. Deploying IoT gateways in smart campuses requires prior planning. Consequently, it is necessary to deploy IoT gateways that address the communication needs of IoT devices in university campuses, such as bandwidth, communication technologies, energy efficiency, etc. The SOFTWAY4IoT project was funded by the National Education and Research Network (RNP) and developed an IoT gateway that can establish communication within an Internet of Things environment. This gateway is virtualized, utilizes software-defined networking (SDN) and fog computing that is integrated with cloud computing, and supports multiple wireless communication technologies [13]. The area coverage of wireless networks is also a challenging problem. The challenges start with the choice of the communication technology that is to be used through to the architectural characteristics of the environment, the number of things or people that access the network simultaneously, the mobility of the people or things that connect to the network, etc. It is possible to find several works that have focused on area coverage in the literature.

We found several works in the literature that focused on area coverage [14,15,16,17,18,19]. Services that require communication infrastructures need a guarantee of the quality of the communication. Distributing the gateways within these environments is a big challenge comprising many factors that are relevant to the positioning and quantification of the devices that aim to meet the presented demand.

In this context, this work proposes a method for the planning and optimization of the deployment of IoT gateways, with the SOFTWAY4IoT IoT gateway as its motivation. Hence, this paper presents three optimization methods for gateway infrastructure planning that aim to minimize the number of required gateways and maximize the coverage area, considering the communication capacity of the data link and the range of the communication technology. A comparison of the models is also presented in order to evaluate the obtained results. The study focused on the LoRa, BLE, and Wi-Fi communication technologies.

The LoRa technology was chosen because it has a long communication range that increases the coverage area, as well as a low energy consumption. On the other hand, the BLE technology opposes LoRa technology in terms of range, as it covers a small area (personal area) but has a similarly low power consumption. Finally, we chose Wi-Fi because it is one of the most popular communication technologies within wireless networking. It has an area coverage that has a larger range than the BLE but a smaller range than the LoRa and has a high power consumption.

However, the determination of how many IoT gateways to use and where to place them in order to maximize the coverage area and minimize the number of gateways is an NP-hard problem, called the WMN node placement problem. The complexity of this problem grows exponentially with small changes in the size of the problem. Therefore, we tested three different hybrid methods to find near optimal solutions [20].

The presented optimization model aimed to minimize the number of required IoT gateways and to position them within the area to be covered, taking into account the size of the area, the range of the communication technology, and the location in which the devices are deployed. The gateway deployment problem was split in two steps: (a) the determination of the number of required gateways by solving a linear programming problem (LPP), which aimed to minimize the gateways quantities; (b) the K-means and particle swarm optimization (PSO) algorithms were employed to establish the gateway positioning.

The use case scenario was a university campus and the chosen areas and the size and characteristics of the environment (indoor/outdoor) are presented in Table 1. We tested larger and smaller areas with different architectural characteristics.

The contributions of this article can be summarized as follows:

- The proposal of an optimization model that uses linear programming for the quantification and positioning of *gateways* to minimize network deployment costs;
- The proposal of an algorithm that uses the K-*means* clustering method to define the positioning of *gateways* within a (predefined) area;
- The proposal of an algorithm that uses the PSO clustering method to define the positioning of gateways within a (predefined) area via two different initialization approaches;
- A comparative evaluation of the methods to establish which method produces the best results for area coverage, using the silhouette coefficient as the metric [21];
- The employment of non-parametric hypothesis testing to verify that the different metaheuristics did not produce results using the same population [22].

This paper is organized as follows. Section 2 presents the related work. Section 3 presents the modeling of the optimization methods that were applied in this study. Section 4 presents the application scenario and Section 5 presents the evaluation of the results. Finally, the final considerations and directions for future work are presented in Section 6.

## 2. Related Works

We considered studies regarding the Internet of Things, intelligent environments, optimization models, clustering methods, and signal propagation, among others, as relevant to the development of this work [23,24,25,26,27,28]. Some studies have analyzed area coverage using equipment for different communication technologies; however, our research considered an intelligent environment using IoT gateways for heterogeneous networks.

The related works were found using the keywords “optimization”, “coverage”, “IoT gateway”, “placement”, and “planning”. These keywords were used to search for works that were related to area coverage and the placement of IoT gateways. We also searched for papers that were related to network signal propagation and smart environments (smart campuses, smart cities, etc.). Initially, the search focused on papers that were published from 2015 onward, but relevant papers were found also from earlier periods. As a search tool, we first used Google Scholar and then we mostly used the IEEE and Springer databases. The searches for related papers were based on the topics of wireless network area planning and coverage, with a greater focus on IoT networks, although this was not restrictive.

A proposal for multi-hop network planning that aimed to minimize hardware (gateways) and operational costs was presented by [14]. Path loss was also considered as an operational cost. The work presented a mathematical optimization model and used three evolutionary algorithms that were based on swarm intelligence [14]. Although the work had similar goals and metrics to this research, there was no application in real space. Our work presents results that were obtained directly from the study environment using three communication technologies and three optimization models.

The model presented by [29] aimed to minimize the latency of communication between fog nodes and gateways. To meet this objective, ref. [29] developed a mathematical formulation that considered the activation of a minimum number of fog nodes. Compared to the proposal presented in this paper, the model in question did not present any study of applied scenarios and adopted empirical parameters in the experiments. There was no quantification of the number of gateways, only a comparison of the executions that varied the numbers.

A study developed by [15] presented a model for application in IoT systems, with a focus on smart cities. The study aimed to reduce the costs of the deployment of an IoT system by considering the use of two gateway models for communication. One of the gateway models communicated with the Internet and the other did not. The presented study used three technologies (Wi-Fi, ZigBee, and RFID) to establish communication between the systems. The work also developed an algorithm to minimize the number of gateways, with the objective of reducing the costs of deployment, and a mechanism to tolerate communication failure.

A smart city scenario integrates several applications of different technologies and may have hundreds of networks using different domains. Each network is coordinated by a coordination device (CD) and each CD needs to transmit its data to the Internet via its own connection that is established using a gateway. To reduce costs, this work proposes two gateway models: an IGW (IoT gateway), which establishes communication using the Internet, and an SSGW (solution-specific gateway), which has no direct communication with the Internet. The gateway presented in our study has features such as SDN usage, virtualization, and integration between the edge and the cloud. These features allow all gateways to communicate with the Internet and can use anything from machines with low processing power, such as a Raspberry, to more robust machines, such as servers with high processing power.

A study on multi-objective planning in WLAN networks was conducted in [16]. The study developed a planning tool that was capable of finding the best position for an access point (AP) and load balance within a WLAN network, which minimized the interference between access points. In this work, a multi-objective evolutionary algorithm with a greedy heuristic was used. The IEEE 802.11 standard was also used, but the study did not address other communication technologies and was restricted to the communication that was established by the access points. For IoT scenarios, heterogeneity is a latent challenge and thus, a study that focuses on IoT needs to consider multi-technology communication. In this regard, our work presents an access point that can integrate multiple communication technologies, i.e., a gateway with multiple communication technologies.

The determination of gateway placements within a network to connect IoT devices is a crucial point when it comes to deployment costs. To solve this problem, ref. [10] presented a solution using integer linear programming (ILP) that minimized the total network costs in relation to deployed devices, while taking into account mandatory quality of service (QoS) requirements. A gateway could be placed anywhere within a given area, but an initial set of candidate positions was considered. Communication took place over multiple hops. To obtain the lowest deployment costs and guarantee the QoS of the fixed transmission range, the specific data rates, end device costs, gateway costs, generated traffic, and the distance between the nodes were used as data. In order to provide QoS, the capacity of the links had to be sufficient to handle multiple simultaneous transmissions.

An optimization approach for gateway deployment in heterogeneous sensor networks was presented by [17]. This study focused on ILP-based optimization and wireless gateway locations. The goal was to minimize the installation costs and maximize the energy efficiency of the wireless sensor network, considering multi-hop coverage and connectivity constraints. Although this study addressed the overheads of sensors that are considered to be critical, multiple hops could demand more time before the message reached its destination.

A gateway placement approach was presented by [18]. This approach aimed to optimize the number of gateways, the average number of mesh routers, and the variations in gateway loads within wireless mesh networks (WMNs). Minimizing the average hop count of the network was one of the objectives since long paths reduce the throughput. The proposed work used two stages to achieve their objectives.

The works of [10,17,18] took into consideration communication using multiple hops. Direct connections between devices and gateways may present higher costs, but the message delivery time tends to be shorter, thereby improving network performance. The particularities of each deployment environment have to be considered, along with issues regarding device density, which involve denser environments or more spacious environments, and other factors. The proposed work considered an intelligent scenario that focused on indoor and outdoor environments and direct communication using software-defined and virtualized gateways. Our optimization model aimed to minimize the number of required gateways as a function of the number of deployed devices to improve the range of the communication technology and the communication capacity of the gateways.

A study developed by [19] addressed the optimal deployment of IoT gateways in smart home environments (smart homes). The work in question solved the optimization problem using the branch and bound method with the goal of minimizing the gateway deployment costs, subject to the constraint that all service areas of the home must be covered. The smart home environment is relatively small compared to a smart campus or smart city. A small environment assumes the use of a single gateway and few devices. For a small environment, the model in question proved to be effective; however, its application in larger environments is necessary to evaluate the effectiveness of the model since there are large areas with high densities of IoT devices.

A literature review article presented by [20] surveyed the optimization approaches that have been implemented to solve the node placement problem in WMNs. In the literature, several WMN node positioning approaches have been proposed. This paper presented a classification that was based on the type of method that was used. The classification was split into four categories: methods that are based on exact approaches, methods that use heuristics, methods that use metaheuristics, and methods that apply hybrid approaches. Additionally, their paper presented a case study using the greedy algorithm (GA), simulated annealing (SA), particle swarm optimization (PSO), and the firefly algorithm (FA) to investigate the impacts of varying the number of mesh clients, the number of mesh routers, and the coverage radius.

Table 2 presents a comparison of the related work, according to network planning. There were works that did not have a focus on IoT networks and others that did not have a focus on gateways, but all of them proposed an optimization model for network planning. All of the works presented map positioning for the devices that established the network communication, although each paper had its own particularities and metrics for mapping. Hence, in this work (and contrary to the related work), our goal was to quantify and position IoT gateways (considering the range of the communication technology that was employed) and the maximum number of devices that could be supported by the gateways.

Section 3 presents the optimization models that were employed in this work and shows the particularities that were adopted in each model.

## 3. Optimization Model for IoT Gateway Planning, Coverage, and Positioning

Several wireless communication technologies are available that can meet the heterogeneous characteristics that guide the Internet of Things paradigm. The application scenarios of this paradigm may include residences, offices, stores, hospitals, industries, universities, cities, etc. The implementation of systems within the context of the Internet of Things demands prior study. In the first instance, this study aimed to analyze the coverage area, the devices that are to be deployed, and the communication technology that would best meet the needs of the environment.

The focus of this work was the planning of IoT gateway deployment within intelligent environments. This research evaluated the signal coverage of three communication technologies (Wi-Fi, LoRa, and BLE) in a smart campus scenario. LoRa is an emerging communication technology with a long range and low power consumption. Wi-Fi is a mid-range technology with a higher power consumption; however, it is widespread in communications and has great relevance to indoor environments. BLE is a short-range communication technology with a low power consumption and personal area coverage.

**Figure 1:**
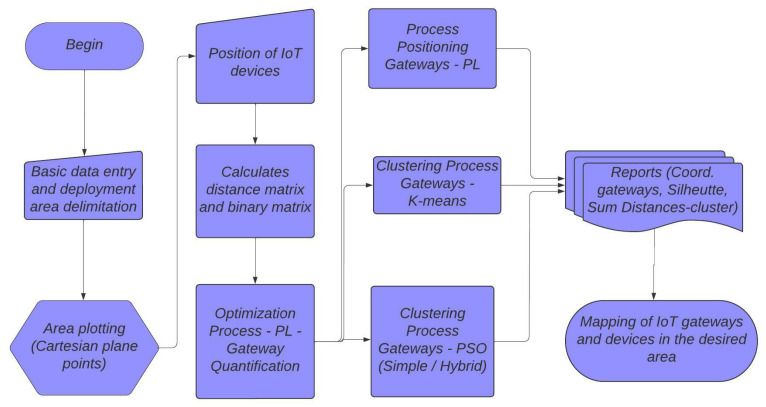
The flowchart of the optimization model.

The proposed work was split into two steps. The first step defined the number of gateways using an optimization model with linear programming. In the second step, the linear programming model, the K-means clustering method, and the PSO method were used to define the positions of the IoT gateways, considering the number of gateways that was defined in the first step. Finally, a comparison was made between the three gateway positioning models, which had the aim of evaluating the results of each model. Figure 1 shows the flowchart of the optimization model.

The candidate points matrix was implemented with the goal of determining the possible installation sites for gateways and IoT devices. We considered the matrix to be a key point for area coverage as its implementation reproduced the floor plan of the scenario, which allowed the algorithm to check each point within the desired area.

The linear programming model developed in this work had a relevance to quantification. The constraints that were associated with the objective allowed for reductions in the deployment costs. The methods, such as K-means and PSO, were used based on the proposed locations of the gateways within the search space that was delimited by the floor plan of the scenario, which is reproduced in this paper in the form of points on a Cartesian plane. These details allowed the quantification and positioning to come closer to the real circumstances of the deployment.

The advantages of the linear programming model over the K-means and PSO models involved the gateway positioning being restricted by the range of the technology; in this case, no device could connect to a gateway when the distance between the points exceeded the range of the technology. On the other hand, the dispersion of devices in relation to the gateways was greater. In the clustering models, there was no restriction on range; however, the degree of dispersion was lower because the devices were positioned considering proximity to the gateways.

The presented models enabled the association of the devices and the experiments were implemented in a real environment with distinct architectures. In this way, it was possible to evaluate their applicability as close to reality as possible. It is noteworthy that the range of the technology needed to consider the study of signal propagation within the deployment scenario. Although this study did not consider the actual coordinates of the deployment of devices, the models were developed with this objective; therefore, when we had the coordinates of the installation site of a device, we inserted them as an input parameter and obtained a result that was even more realistic.

### 3.1. Gateway Quantification and Positioning Using Linear Programming

The planning and optimization of IoT gateway deployment for communication networks aimed to calculate the minimum gateway quantity that is required to cover a defined area and the number of IoT devices per gateway. For the quantification, the range of the employed wireless communication technology and the gateway communication link capacity were considered. The ultimate goal was to minimize the number of gateways while maximizing the coverage for devices within the desired area.

The area coverage was calculated based on the signal propagation of the communication technology. The signal propagation of each communication technology had distinct characteristics and also varied with respect to the propagation environment (indoor/outdoor). The signal propagation models allowed the range of the communication technology to be abstracted as a function of path loss.

**Figure 2:**
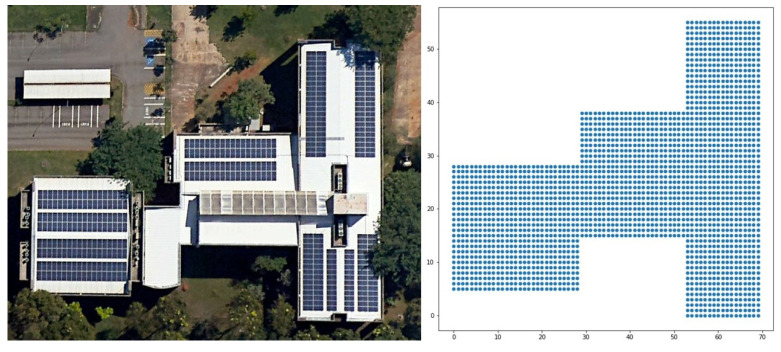
An example of area mapping using the Institute of Informatics, UFG.

The coverage area was an input parameter for the optimization model. Using Google Earth as a tool (available online: https://www.google.com.br/intl/pt-BR/earth/ (accessed on 10 May 2021)), it was possible to ascertain the measurements (dimensions) of the desired area. This area needed to be delimited and the possible locations of the devices and gateways needed to be plotted. In the optimization model, the points were plotted according to the coordinates of a Cartesian plane, as shown in Figure 2. In this case, we obtained an area of 2425 m^2^ that was considered for the possible device locations. Each m^2^ was one positioned point (i.e., a possible point in the desired area), totaling 2425 possible IoT device installation points.

Each pair of coordinates for the plotted points in the specific area was used as an input parameter for the linear programming-based optimization model. The proposed model chose the installation points of the IoT devices randomly using a seed, but they could also be defined according to the reality of their deployment (i.e., their real positions). When using the real positions, it was necessary to have the coordinates of the location in which the device was to be deployed.

As input parameters, we also used the range of the communication technology, the number of devices, the gateway data link capacity, and the IoT device link demands. The presented metaheuristic quantified the IoT gateways within the desired area, considering the coverage of the devices that were deployed in that area. Table 3 presents the parameters that were used for the optimization model that was based on linear programming. Considering the goal of minimizing the number of gateways, the decision variables were defined first. Then, we obtained:

- $X_{i,j}$ as the matrix that associated a device to a gateway;
- $Y_{i}$ as the vector that received the gateways that were activated to meet the demand of the devices.

Given the coordinates of the IoT devices ($D_{i,j}$), the optimization model associated each device with a gateway ($Y_{i}$). The gateway had to be within the range of the technology (*r*). This association occurred via the decision variables. Each device was assigned to a gateway but each defined gateway could have multiple devices associated with it. The model used these constraints to make associations that obeyed the necessary criteria in order to meet the proposed objective. The objective of the optimization model is represented by Equation (1):

(1) $$min\;\sum_{i=1}^{n}\;Y_{i}$$

where $Y_{i}$ represents the active gateways. Equation (1) represents the objective of minimizing the number of gateways ($Y_{i}$). Note that each $D_{i,j}$ is a candidate gateway point. Using this logic, a gateway could always be activated at one of the points $D_{i,j}$, which was equivalent to an IoT device point.

Constraints were defined to achieve the goal of ensuring coverage for the IoT devices using the lowest number of gateways. As constraints, we used:

(2) $$\sum_{j=1}^{n}\;X_{i,j}\;=\;1,\;i=1,\cdots ,n.$$

(3) $$X_{i,j}\;\leq \;Mativ_{i,j}\;*\;Y_{i}\;\;i,j=1,\cdots ,n$$

(4) $$\sum_{j=1}^{n}\;X_{i,j}\;*\;c\;-\;cb\;\leq \;0$$

(5) $$X_{i,j}\;\geq \;0,\;i,j=1,\cdots ,n$$

(6) $$Y_{i}\;\geq \;0,\;i,j=1,\cdots ,n$$

The restrictions caused by Equations (2) and (3) aimed to associate a device with a single gateway. Equation (4) limited the number of devices per gateway, considering the link transmission capacity, and did not allow for the exceedance of the defined maximum transmission capacity. In this case, when the maximum capacity was exceeded, another gateway was activated to meet the required demands. The constraints caused by Equations (5) and (6) were non-negative constraints.

Running the optimization model produced the active gateways ($Y_{i}$) and the associations between the devices and their corresponding gateways ($X_{i,j})$ as solutions. From those results, a graph could be plotted using the plant model represented by Figure 2, which showed the positions of the gateways and the respective IoT devices, thus forming a cluster.

### 3.2. Gateway Placement Using the K-Means Clustering Algorithm

The K-means clustering method is used in data mining [31]. This method aims to partition *n* observations into *k* clusters. In this research, the K-means algorithm was used to define the gateway placements and the distribution of devices per gateway.

The definition of a cluster quantity in the K-means approach was used as an input parameter. The input parameters that were used in this model are presented in Table 4.

The K-means method uses the distance between points for clustering and attempts to separate samples into *n* clusters of equal variance by minimizing a criterion, which is known as inertia, or the sum of squares within the cluster according to Equation (7):

(7) $$\sum_{j=1}^{n}\;(\|x_{j}\;-\;\mu \;{\|}^{2})$$

The proposed algorithm divided a set of *n* IoT devices into *k* groups. The center of the groups (centroid) represents the position of the IoT gateway and $D_{i,j}$ represents the coordinates/position of the IoT devices within the defined area $A_{i,j}$. In the performed experiments, the devices ($D_{i,j}$) were defined randomly using points within the desired area $\left(A_{i,j}\right)$. It is worth pointing out that they were defined randomly because it was an experiment. The points of a device must have its position defined as coordinates on a Cartesian plane in a real situation.

**Figure 3:**
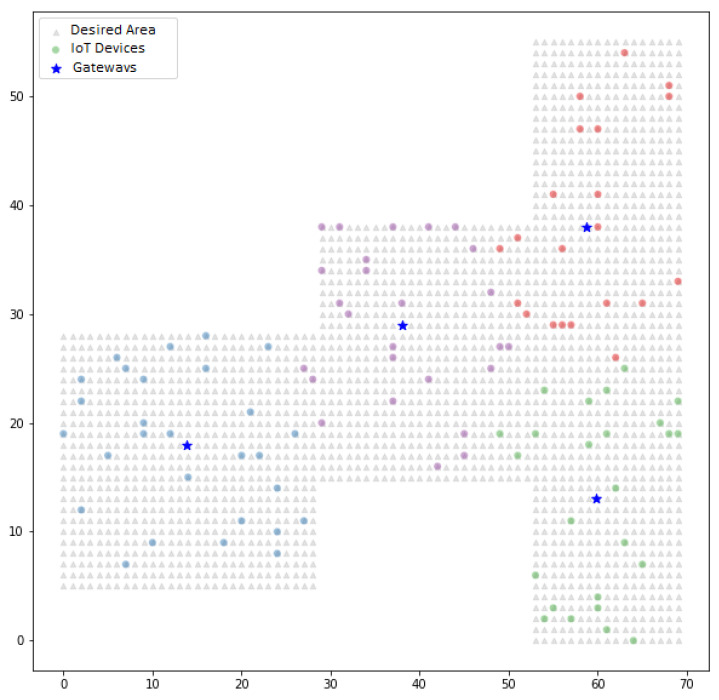
An example of the results of device and gateway placement using the K-means method.

As the number of gateways was defined by the results (C.f. Figure 3) of the first stage of execution, it was understood that the areas and positions of the devices had to be the same for the execution of the second stage (clustering). In order to retain the same position of the devices, the same seed was used for all execution steps. In this way, the devices were randomly chosen based on the same seed.

The K-means algorithm selected the position of the centroids randomly. The input K-means parameter from the scikit-learn library was applied to initialize the centroids more intelligently in order to speed up convergence. Using this parameter, the K-means algorithm randomly positioned a first centroid, then the other centroids were positioned so that they were as far away from each other as possible. This initial positioning helped the algorithm to converge faster.

Algorithm 1 positioned all gateways (*c*) and assigned to them the nearest devices ($D_{i,j}$), so that all devices were associated with a gateway. The process of assigning and reallocating centroids was repeated until the positions of the centroids were stable (convergence). The goal was make the sum of the distances between the devices ($D_{i,j}$) and the gateways (*c*) as small as possible.
**Algorithm 1: **The K-means pseudocode.**input**: $N,k,A_{i,j},D_{i,j}$ 
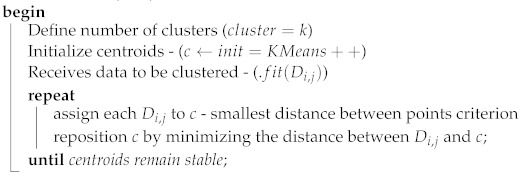
**output**: Grouping the devices to the respective gateway forming the clusters **output**: Reports with metrics related to the clustering process

### 3.3. Positioning of Gateways Using the PSO Optimization Algorithm

The particle swarm optimization model is so named because it is an evolutionary algorithm that arose from studies on algorithms that modeled the social behavior of animals (e.g., bird flocking and bee swarming behavior).

It had the same parameters as the K-means method: the number of gateways (*c*), the number of devices (*n*), the desired area ($A_{i,j}$), and the device positions ($D_{i,j}$). The number of particles (*p*) was a unique parameter for the PSO model. The number of particles was then defined as an input parameter. Each particle was a vector that contained the same number of positions as gateways, so we obtained $P_{i}=\left(P_{1},P_{2},\cdots ,P_{c}\right)$.

Each particle in the population represented one possible solution. The particles moved around the search space, looking for the position that best met the objective function (fitness function). These particles moved at a certain speed (*v*) and had a “memory” that saved their best position (pbest). A “collective memory” was considered for the swarm and represented the best global position that was reached (gbest). Table 5 presents a summary of the variables that were applied to the PSO model that was developed in this research.

The initialization of the particles took place randomly within the search area. Once initialized, the gbest was updated with the positions of the particles. This model positioned the gateways considering the best global position of the particles. Following the flow, the particle velocity was updated, as presented in Equations (8) and (9), which updated the particle positions within a given iteration:

(8) $$v_{i}^{\left(t+1\right)}=w^{\left(t\right)}v_{i}^{\left(t\right)}+c_{1}r_{1}\left(pbest_{i}^{\left(t\right)}-p_{i}^{\left(t\right)}\right)+c_{2}r_{2}*\left(gbest_{i}^{\left(t\right)}-p_{i}^{\left(t\right)}\right)$$

where $v_{i}^{\left(t+1\right)}$ is the velocity to be updated, $w^{\left(t\right)}$ is the inertia factor at iteration *t*, $c_{1}$ and $c_{2}$ are the cognitive and social coefficients, respectively, $r_{1}$ and $r_{2}$ are the random values between 0 and 1, and $pbest_{i}^{\left(t\right)}$ is the best local point and best global point at iteration *t*.

(9) $$p_{i}^{\left(t+1\right)}=p_{i}^{\left(t\right)}+v_{i}^{\left(t+1\right)}$$

where $p_{i}^{\left(t+1\right)}$ represents the new position of the particle, $p_{i}^{\left(t\right)}$ is the current position of the particle, and $v_{i}^{\left(t+1\right)}$ is the new velocity, as calculated by Equation (8).

The inertia factor (*w*) contributes to particle convergence. Larger values of *w* contribute to a global search that explores new areas of the search space. As values of *w* decrease, they favor local searches, which is interesting when the particles are close to a good solution. The presented PSO model applied a linear variation of the inertia factor over the number of iterations, as shown in Equation (10):

(10) $$w^{\left(t\right)}=w_{initial}-\left(w_{initial}-w_{final}\right)\frac{t}{mi}$$

where w(t) is the inertia at iteration *t*, $w_{initial}$ is the initial inertia, $w_{final}$ is the inertia for the last iteration, *t* is the current iteration, and mi is the maximum number of iterations.

The coefficients $c_{1},c_{2}$, and $w_{initial}/w_{final}$ were input parameters, while the coefficients $r_{1}$ and $r_{2}$ were random values between 0 and 1 that were calculated by the system.

The PSO updated the positions of the particles until it reached the maximum number of iterations, as presented in Algorithm 2. The particles converged to a point that was considered to be the best global position.
**Algorithm 2: **The PSO pseudocode.
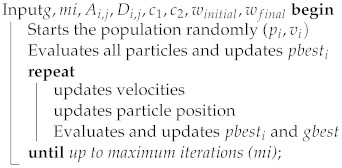
**output**: Grouping of the devices to the respective gateway forming the clusters **output**: Reports with metrics related to the clustering process

## 4. Description of the Application Environment

The proposed models were applied in a case study in order to evaluate their results. The models allowed for the quantitative measurement of gateway positioning within the coverage area.

From the point of view of the deployment of IoT devices in a smart campus, several possibilities are envisioned to meet diverse demands, such as: monitoring the environment, humidity, gas levels, and temperature; monitoring parking lots; access monitoring via video cameras; and intelligent lighting control. All of these examples contribute toward facilitating daily activities and improving the quality of life of the academic community.

This section discusses the case study scenario and presents the characteristics and parameters that were explored.

### Scenario Description

Smart campus deployment is marked by the heterogeneity of services, assuming the heterogeneity of the technologies that can be used to deploy services within the environment. The application of the optimization model to three communication technologies was proposed in this work: one with a personal area coverage with low power consumption (BLE); one with local and commonly known area coverage (Wi-Fi); and one with long range areas with low power consumption (LoRa).

Each technology has a different range. It has also been observed in published studies in the literature that each environment has objects and structures that interfere with the propagation of wireless network signals. For the case study, we searched the literature for signal propagation studies regarding BLE, Wi-Fi, and LoRa technologies. The signal propagation studies were based on the received signal strength indication (RSSI). From the measurement of the RSSI and the application of signal propagation models, it was possible to determine the range of the technology. The RSSI for the range radii of the tested technologies was below −90 dBm. In this work, we adopted the range measurements according to Table 6.

**Figure 4:**
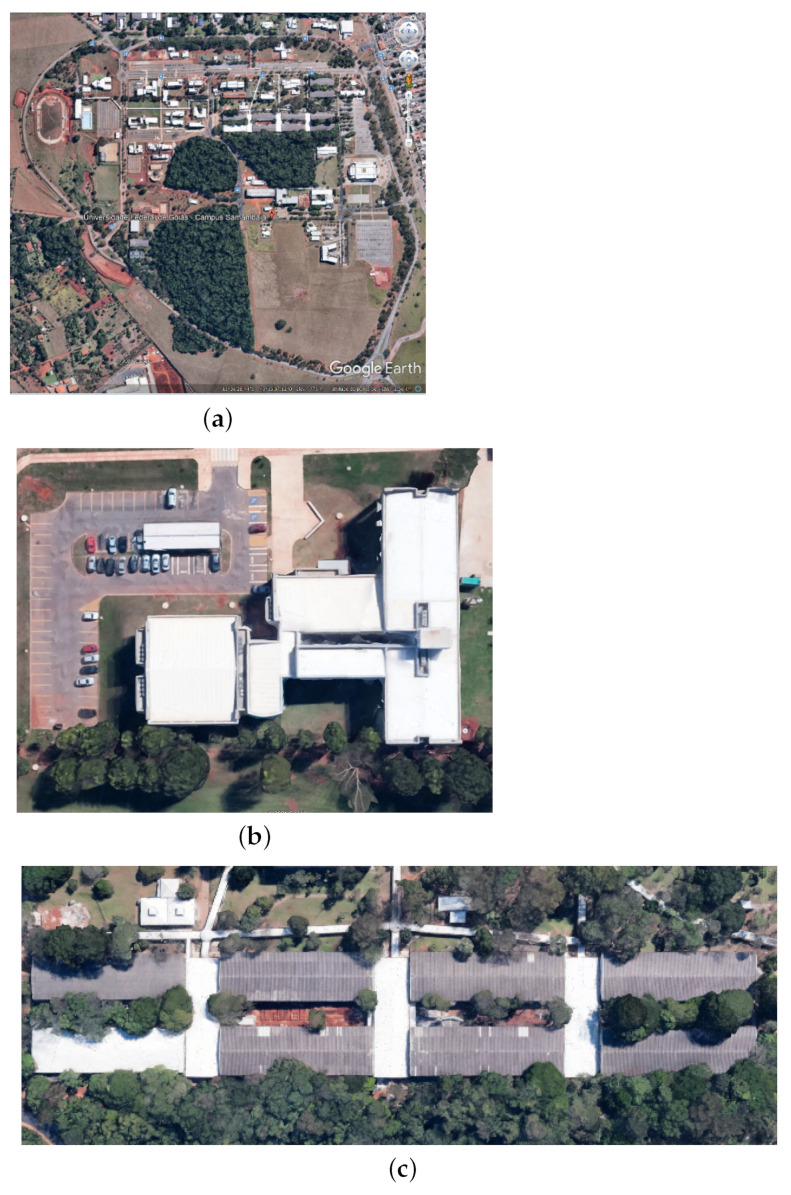
The Samambaia Campus and departments of UFG: (**a**) the Samambaia Campus; (**b**) the Institute of Informatics; (**c**) the academic blocks.

The case study was applied in a smart campus scenario. As an outdoor environment, we could use the whole area of the Samambaia Campus at the Universidade Federeal de Goiás (UFG). In this work, we considered an area of 761,380 m${}^{2}$, excluding the woods and unbuilt areas (Figure 4a). The indoor environment was the INF with 2425 m${}^{2}$ (Figure 4b) and an area of academic blocks with 26,664 m${}^{2}$ (Figure 4c), comprising the Institute of Biological Sciences (ICB I and II), Institute of Chemistry (IQ), Institute of Physics (IF), Faculty of Philosophy (FAFI), Faculty of History (FH), and Faculty of Communication and Librarianship (FACOMB). The measurements were taken using Google Earth.

**Figure 5:**
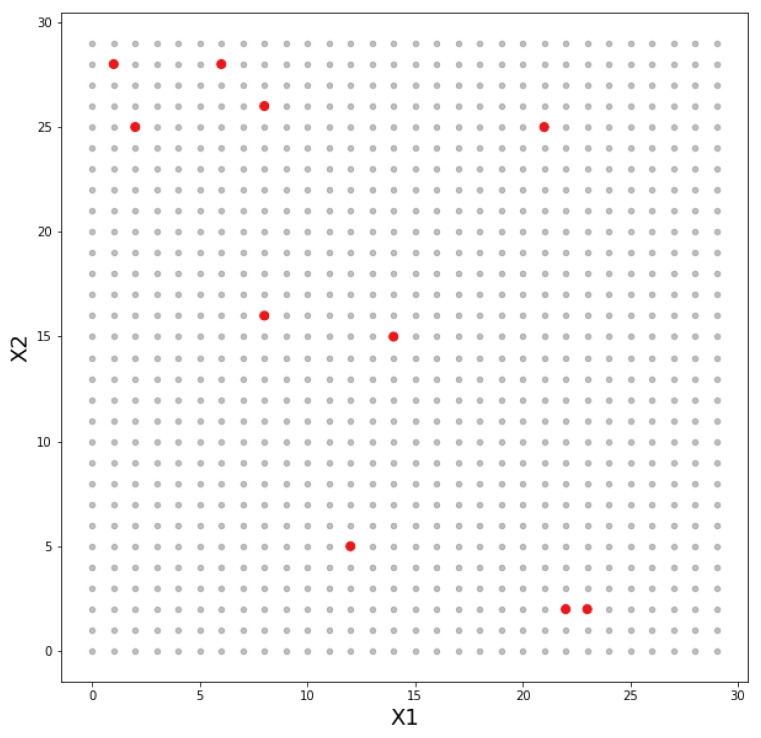
An example of how IoT points and devices are plotted within the desired area by generating an *x*,*y* coordinate matrix.

The presented optimization model recognized the desired area by defining points on a Cartesian plane (*x* and *y* coordinates). These coordinates indicated where an IoT device or gateway could be deployed. Figure 5 shows an example of plotting the points on a Cartesian plane, with an area of 100 m${}^{2}$ containing 10 deployed IoT devices. The points in gray are all possible deployment points, while the points in red represent the deployed IoT devices.

A real application of the optimization model should have the coordinate pair of each device as an input parameter. For the purposes of these experiments, the coordinates of the IoT devices were set randomly among the points in the desired area. Once the device coordinates were defined, a distance matrix between the device points (see Table 7) was calculated, which was the basis for the generation of the gateway activation matrix (see Table 8).

The distance matrix was generated from the calculation of the Euclidean distance between one point and all of the other points. In the example presented in Table 7 and Table 8, 10 IoT devices and a range of 25 m were considered. The activation matrix was binary and was generated from the distance matrix. When the distance was less than the range, it was assigned the value of 1 (one); when the distance was greater than the range, it was assigned the value of 0 (zero).

The parameters that were used in the proposed models were presented in Section 3. In particular, the PSO model used the following values as the initial values for the execution of the experiments: $w_{initial}=0.9$, $w_{final}=0.3$, np=20, mi=150, c1=2.1, and c2=1.9.

In Section 5, the results of the experiments and an analysis of the comparison of the three metaheuristics are presented.

## 5. Presentation and Evaluation of Results

In order to achieve the proposed goal, the research aimed to present three algorithms that focused on planning and optimizing the deployment of IoT gateways. The first was based on linear programming, which aimed to quantify and position the gateways. The second and the third employed clustering using the K-means and PSO algorithms, which aimed to position the gateways.

The research relied on the case study to refine the results; then, the results were compared to evaluate the three methods that were applied to the case study. The initial parameters were set. A floor plan of the studied environment with the plots of the relevant points on a Cartesian plane and the definition of the points of the installed IoT devices were used as input parameters, as well as the range of the technologies and the capacity of the data links. The same parameters were applied to the three metaheuristics. After the experiments, the results were compared to analyze the positioning of the gateways and the distribution of the devices. Three environments were used in the case study (indoor and outdoor environments) to evaluate the behavior of the metaheuristics and the obtained results.

### 5.1. LP Optimization Model: Gateway Quantification and Positioning

The experiments presented in this section aimed to evaluate the optimization model that was based on linear programming. Table 9 presents the results of the run, considering the defined values.

The first experiment took into account the deployment of 100 or 300 devices for each communication technology. It was observed that for the BLE technology, with a demand of 0% or 3% of the data link for each device, we obtained the same results of 35 and 37 gateways, respectively. The value remained the same because the range was the parameter that prevailed in the definition of the quantity of gateways. The opposite was observed with the Wi-Fi and LoRa technologies. When Wi-Fi technology consumed 30% of the link demand, it was necessary to have 4 gateways to serve 100 devices and 10 gateways to serve 300 devices; the same occurred with LoRa. This equality between LoRa and Wi-Fi occurred because the same bandwidth consumption was defined for both technologies. Thus, when analyzing the same technologies with 0% bandwidth consumption, it was observed that Wi-Fi communication required 1 less gateway to serve 100 devices and 7 less gateways to serve 300 devices. It was also found that LoRa demanded 3 fewer gateways to serve 100 devices and 9 fewer gateways to serve 300 devices. This variation in the number of required gateways proved that the presented optimization model considered the range and the demand for the data links.

The main goal of this optimization model was to quantify the gateways. The model was proposed based on gateway activation criteria as a function of the Euclidean distance between the IoT devices and the corresponding gateway and the transmission capacity of the communication link to the gateway. When the gateway was activated, it was assigned the devices that were within its range, while obeying the established constraints. The process was repeated until the smallest possible number of gateways was found to serve all of the deployed devices.

**Figure 6:**
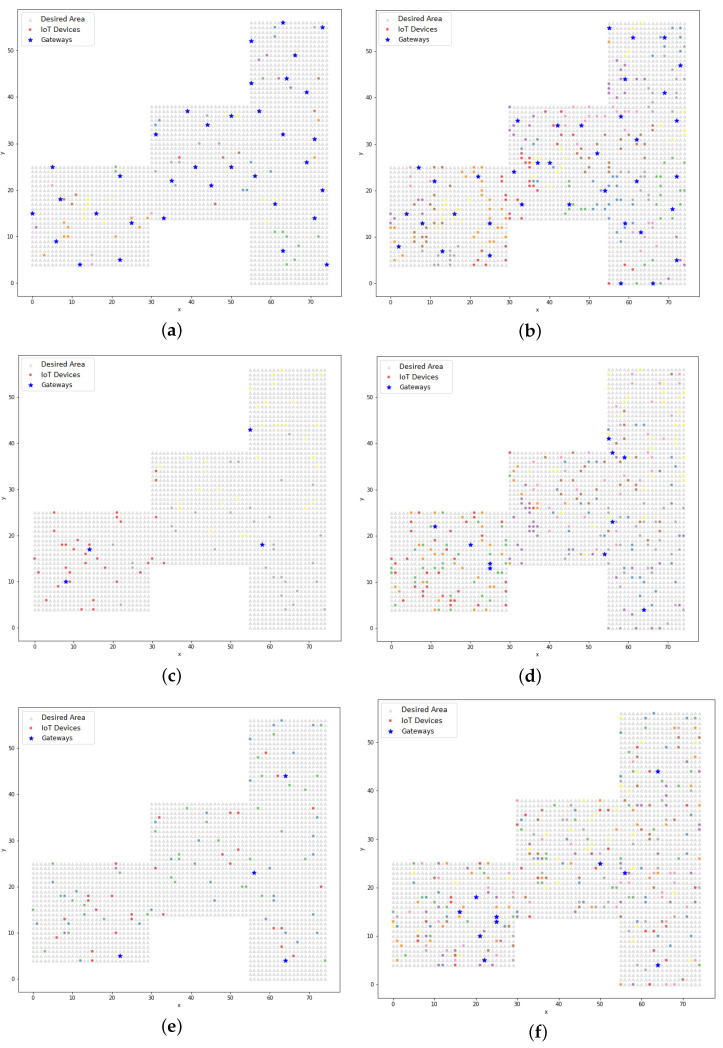
The plots of the devices and their respective gateways with a3% data link consumption (LP method): (**a**) BLE technology with 100 devices and 35 gateways; (**b**) BLE technology with 300 devices and 37 gateways; (**c**) Wi-Fi technology with 100 devices and 4 gateways; (**d**) Wi-Fi technology with 300 devices and 10 gateways; (**e**) LoRA technology with 100 devices and 4 gateways; (**f**) LoRA technology with 300 devices and 10 gateways.

The association between the devices and the gateway allowed for a map of their positions to be plotted, as shown in Figure 6. In the LP model, the association was defined considering the range of the communication technology, whereas in the clustering process, the association was based on the proximity of the devices to the gateway.

The association between the devices and the corresponding gateway is represented by the alternating colors of the devices in the graph. These figures were equivalent to the execution, the results of which are shown in Table 9. A device could be at different ends of the defined area, but when it was within the range of coverage, it could be associated with the gateway. There are two columns called “Silhouette” in Table 9: this metric is a coefficient that indicates how cohesive a data cluster is. This coefficient ranged from −1 to 1 in this study. The closer to 1 the coefficient, the more cohesive the data; the closer to −1, the wider the dispersion.

As mentioned above, the distribution of the gateway devices that is represented by Figure 6e had the lowest Silhouette coefficient, indicating that the distribution was the least cohesive. This coefficient proved that dispersion when distributing the devices was significant.

Although a graphical representation of the positions of the devices/gateways with a bandwidth consumption that was equal to zero is not presented, it was observed that the distribution was more cohesive, according to the silhouette coefficients that are seen in Table 9. The silhouette coefficient can only be calculated when there are two or more clusters, thus justifying the absence of this coefficient from some of the presented results.

The optimization model could be applied to different areas, each with its own architectural characteristics. In this work, it was applied in three areas: two indoor areas (INF and the UFG academic blocks) and one outdoor area (the entire Samambaia Campus area of the UFG). The measurements of these areas are presented in Table 1 and the ranges of the technologies are presented in Table 6.

**Figure 7:**
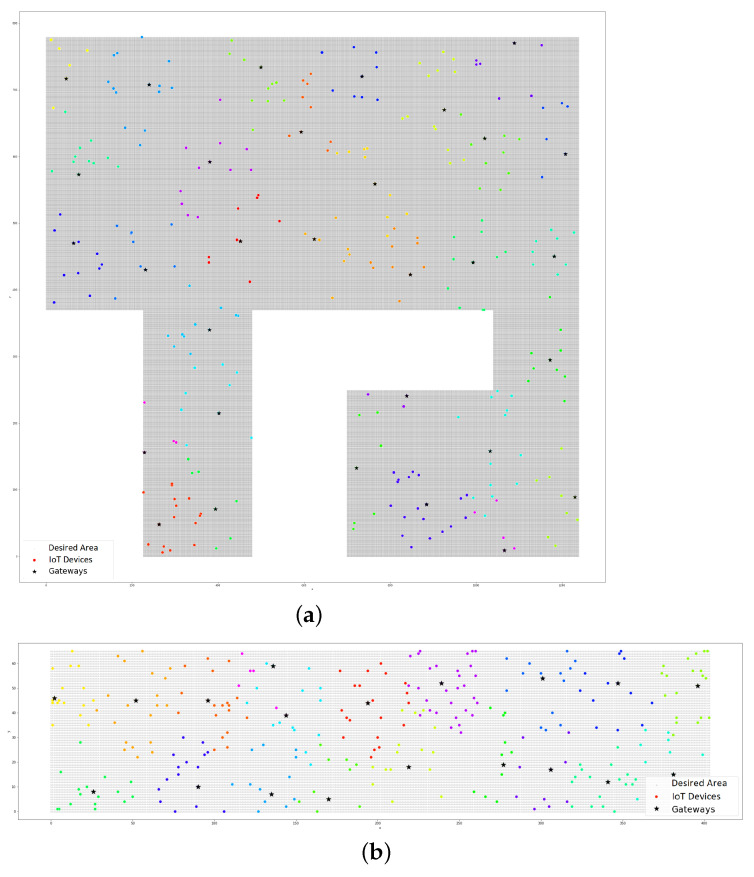
The plots of the devices and their gateways (Wi-Fi technology): (**a**) the Samambaia Campus with 300 devices (outdoor environment); (**b**) the academic blocks with 300 devices (indoor environment).

The experiments that were performed in other areas produced the results that are shown in Figure 7a,b. In these experiments, we tested the Samambaia Campus with 300 IoT devices and 31 gateways to serve a 100-m range (Wi-Fi outdoor environment) with a 3% bandwidth consumption and we tested 300 IoT devices and 19 gateways in the academic blocks of the Samambaia Campus with the same bandwidth consumption and a 25-m range (indoor environment).

**Figure 8:**
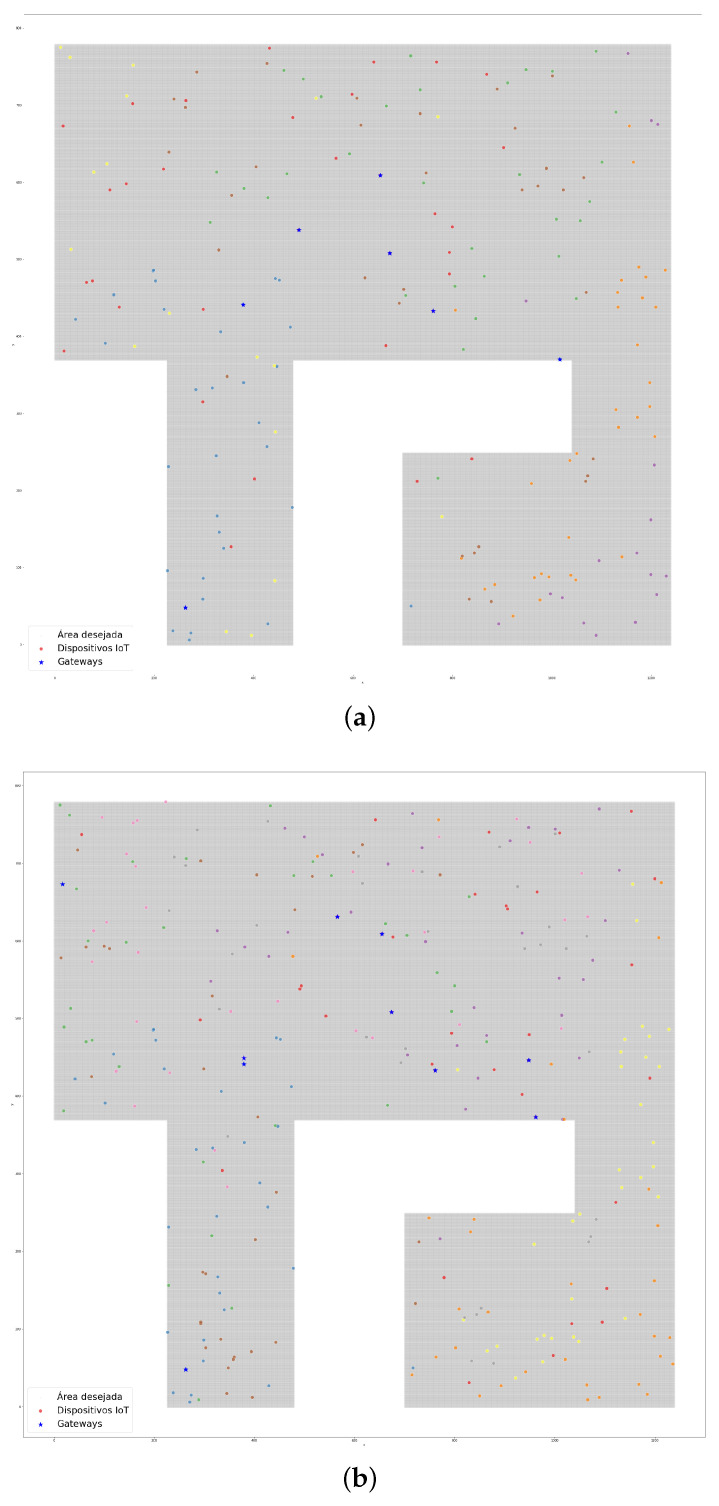
The plots of the devices and their gateways (LoRa technology; outdoor environment): (**a**) the Samambaia Campus with 200 devices and 7 gateways; (**b**) the Samambaia Campus 300 devices and 10 gateways.

The LoRa communication technology has as a remarkable long-distance range. Taking this characteristic into account, we conducted the experiments at the Samambaia Campus (outdoor environment). We considered a range of 500 m for the LoRa technology in the outdoor environment. The distribution of the gateways and IoT devices are presented in Figure 8. In the scenario in question, 7 gateways were required for 200 devices with 3% bandwidth consumption, as shown in Figure 8a. Figure 8b presents the results for the same scenario but for 300 devices with 3% bandwidth consumption, which demanded 10 gateways.

Table 10 presents a report of the execution of the optimization model when applied to the academic blocks of the Samambaia Campus. Table 10 shows the number of IoT devices that were served by each gateway. Taking into account the bandwidth consumption of 3%, each gateway could serve a maximum of 33 devices. In the presented results, the number of clusters/gateways that served the most IoT devices was the 17, serving 31 devices. The second column of the table shows the largest calculated distance between a device and the corresponding gateway within the respective cluster. It can be seen that no value in that column exceeded the range of the technology (25 m).

The results that are presented in these three scenarios show that the optimization model could be applied in different scenarios by changing the parameters according to the desired situation.

**Figure 9:**
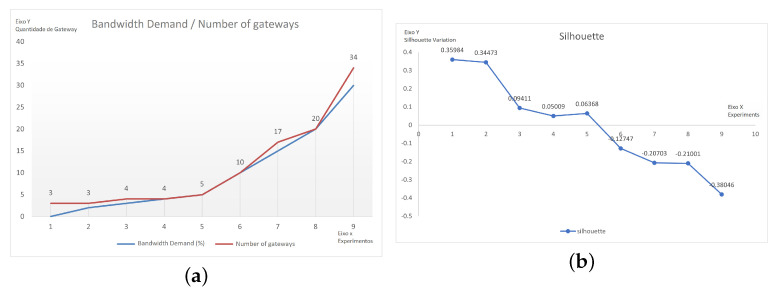
Graphs showing the results of the experiments with variations in the demand for data link consumption: (**a**) the data link consumption × quantity of gateways; (**b**) the silhouette coefficient.

Some of the experiments that were applied to the INF scenario considered 100 devices that were communicating via Wi-Fi technology with varying demands for link capacity. The results are presented in Table 11. It can be seen that an increase in demand for bandwidth consumption caused an increase in the number of gateways, so the dispersion of the devices within the clusters was noticeable. Evidence for the dispersion can also be seen in the variations in the silhouette coefficient. Figure 9a shows the increases in the bandwidth demand and the number of gateways and Figure 9b shows the decrease in the coefficient.

The obtained results confirmed that varying the range of the communication technology and the demand for data link consumption directly influenced the required number of gateways.

It was also observed that the distribution of gateways and devices using this optimization model was not achieved using a clustering technique. In Section 5.2 and Section 5.3, the results from the placement of gateways using the K-means clustering model and the PSO model are presented, respectively.

### 5.2. K-Means Model: Clustering and Gateway Placement

The K-means clustering model uses Euclidean distance as a metric to divide the devices into *n* groups. The center of each group (centroid) is considered to be a gateway. This section presents the results of the application of the clustering model, taking into account the parameters presented in Table 9, which show the comparison between the three communication technologies (Wi-Fi, BLE, and LoRa) when varying the number of devices that were distributed in the area.

Table 12 presents the silhouette coefficients after running the K-means model. The results shown by the silhouette coefficients were good, proving the cohesion of the clustering. A comparative analysis between the silhouette coefficients from the K-means model and those from the other clustering models is presented in Section 5.4.

**Figure 10:**
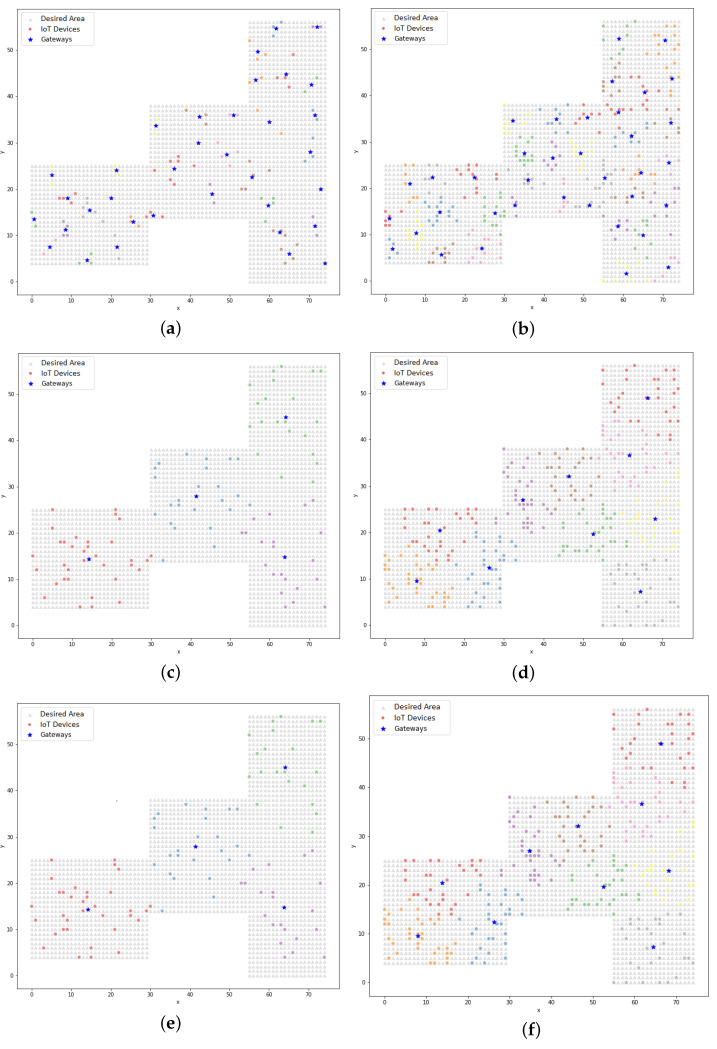
The clustering of IoT devices and their respective gateways using the K-means model: (**a**) BLE technology with 100 devices and 35 gateways; (**b**) BLE technology with 300 devices and 37 gateways; (**c**) Wi-Fi technology with 100 devices and 4 gateways; (**d**) Wi-Fi technology with 300 devices and 10 gateways; (**e**) LoRa technology with 100 devices and 4 gateways; (**f**) LoRa technology with 300 devices and 10 gateways.

Graphically, it can be observed in Figure 10 that the clusters were formed by the devices that were close to the centroids, unlike the dispersion that was observed in Figure 6. The dispersion was verified by the silhouette coefficient. Analyzing the resulting positions from the K-means model using the LP model, it could be seen that the K-means model treated the gateways as the center of the cluster and, in this case, the radius of the technology was not a limiting factor in the clustering process (unlike the treatment from the LP model). In Section 5.3, the PSO model is addressed and in Section 5.4, a comparative analysis between the proposed models is presented, which found that in some cases with the clustering model, the distance between a device and a gateway could be greater than the range of the technology.

### 5.3. PSO Model: Clustering and Gateway Placement

The PSO positioning model was inspired by the collective movement of animals, such as a flock of birds or a school of fish. The model proposed in this research had two approaches: a simple PSO and a hybrid PSO. The difference between the approaches was in the initialization of the particles. In the simple approach, the particles initialized randomly within the search space and, depending on their initial position, moved during each iteration in search of the best global position. In the hybrid approach, the initialization of the particles occurred using the initialization model that was adopted in the K-means algorithm, called K-means++. The results of the two approaches are presented in Section 5.3.1 and Section 5.3.2, respectively.

#### 5.3.1. Simple PSO

As with the K-means clustering model, Table 13 demonstrates the silhouette coefficient values from the simple PSO model. The variations in these coefficients came from variations in the adopted parameters, but they still showed good results regarding the cohesion of the clusters.

**Figure 11:**
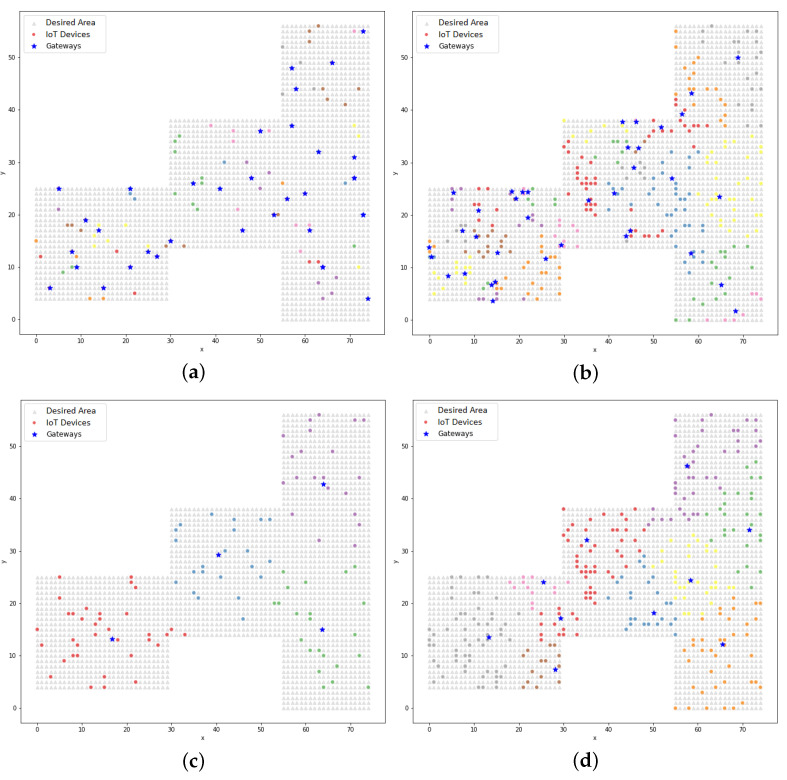

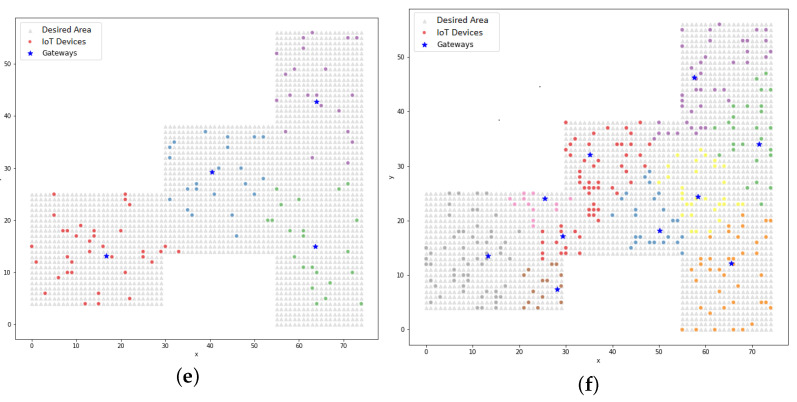
The clustering of IoT devices and their respective gateways using the PSO model (simple approach): (**a**) BLE technology with 100 devices and 35 gateways; (**b**) BLE technology with 300 devices and 37 gateways; (**c**) Wi-Fi technology with 100 devices and 4 gateways; (**d**) Wi-Fi technology with 300 devices and 10 gateways; (**e**) LoRa technology with 100 devices and 4 gateways; (**f**) LoRa technology with 300 devices and 10 gateways.

Figure 11 shows the distribution of gateways and IoT devices that was achieved using the PSO model (simple approach). In this model, it could be seen that the distribution of gateways allowed for a higher concentration in certain regions of the search area as well as other sparser regions, as shown in Figure 11b. This occurred because of the randomness of the initial positions of the particles, which interfered with the positioning results across the iterations. The initial positions could be concentrated in one region and by updating the positions of the particles using the velocity and the cognitive and social factors, this positioning was achieved.

#### 5.3.2. Hybrid PSO

The hybrid PSO approach was a clustering model that aimed to improve the initial positioning of the particles using the initialization method that was adopted in the K-means model. The initialization was used in the first iteration and forced the centroids to be further away from each other.

When comparing the silhouette coefficients from the simple PSO model (Table 13) to those from the hybrid PSO model (Table 14), it could be seen that the larger coefficients were always from the hybrid approach, which confirmed that the better initial positioning of the particles resulted in better final positions.

**Figure 12:**
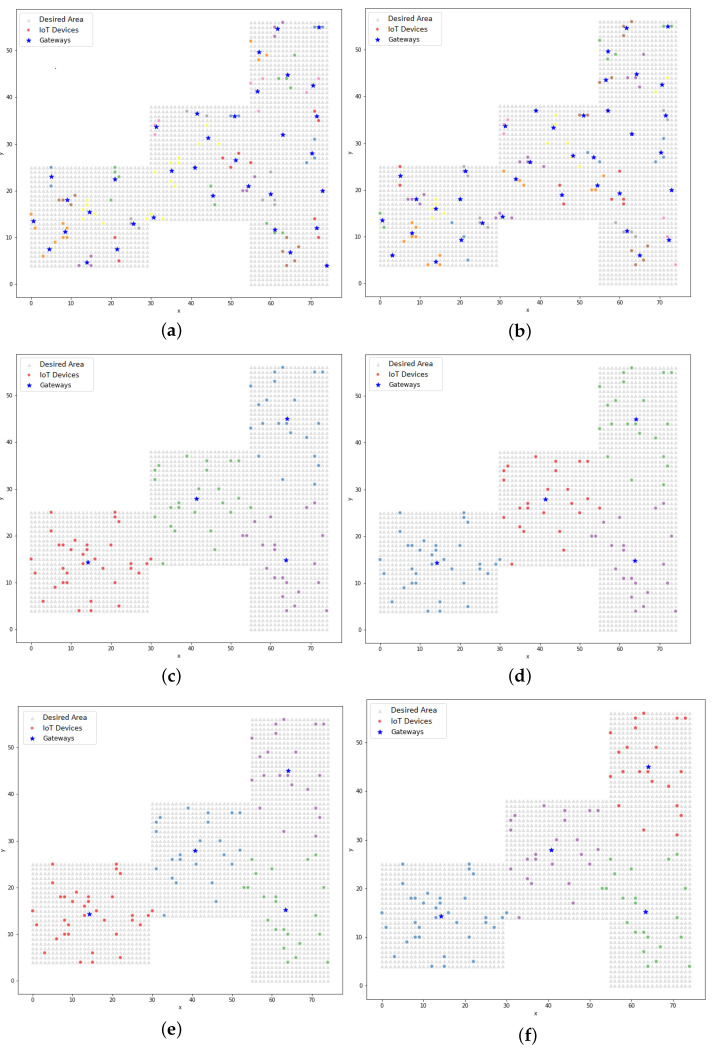
The clustering of IoT devices and their respective gateways using the PSO model (hybrid approach): (**a**) BLE technology with 100 devices and 35 gateways; (**b**) BLE technology with 300 devices and 37 gateways; (**c**) Wi-Fi technology with 100 devices and 4 gateways; (**d**) Wi-Fi technology with 300 devices and 10 gateways; (**e**) LoRa technology with 100 devices and 4 gateways; (**f**) LoRa technology with 300 devices and 10 gateways.

The distribution of IoT devices and their corresponding gateways that resulted from the hybrid PSO model is presented in Figure 12. When comparing these results to those from the simple PSO model, it could be visually observed that the gateway positioning in the hybrid PSO model always maintained a better distribution within the desired area, especially when looking at Figures b and Figure 12b.

**Figure 13:**
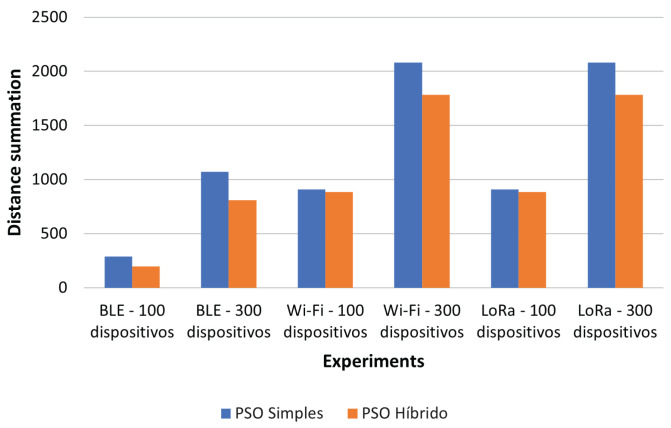
The comparison of the total distances between the IoT devices and their gateways using the simple and hybrid PSO models.

Figure 13 presents a comparison of the two PSO approaches. The comparison was carried out by summing the calculated Euclidean distances between the IoT devices and their respective gateways. The larger the sum of the distances, the less cohesive the cluster. In all of the presented runs, the hybrid PSO model had smaller sums of the distances, thereby proving that the clustering in the hybrid model was better.

The experiments presented in Section 5.1–Section 5.3 were mainly aimed at testing how well the models worked. From those experiments, it was proved that it was possible to quantify, assign, and cluster the IoT devices and their gateways. A comparative analysis was performed to evaluate the four models: LP, K-means, simple PSO, and hybrid PSO. The analysis is described in Section 5.4.

### 5.4. Discussion: Comparative Analysis and Evaluation of Presented Results

The present work proposed three optimization models. The LP model aimed to quantify and position gateways by assigning IoT devices to a respective gateway within the desired area as a function of the radius and data link demand. The K-means model and the PSO model positioned the gateways according to the number that was defined by the LP model and distributed the IoT devices by considering the Euclidean distance between them.

For the three models in question, metrics could be abstracted for a comparative analysis. The silhouette coefficient was one of the metrics considered. Another factor taken into consideration was the sum of the Euclidean distances between the devices and their respective gateways. This result was calculated for each cluster and then totaled.

The models could be applied in several scenarios. In this work, we considered three scenarios: INF, the Samambaia Campus, and the academic blocks. The architecture of the buildings does not represent a regular geometric figure, which aroused interest in analyzing the behavior of the models.

Since we tested different scenarios, Table 15 presents the comparisons between the models within the same scenario. It can be seen that the total distances and the best silhouette coefficients were found by the K-means and hybrid PSO models. The K-means model defined the position of the centroids and, at each iteration, repositioned them to the center of the cluster. In this way, the clusters always had their gateways in the center. The hybrid PSO model defined the initial positions of the particles using the K-means initialization technique and led the particles to better initial positions, which was reflected throughout each iteration, thereby proving that once initialized well, particle movements tend to finalize well. The LP model showed good summations and silhouette coefficients, proving that they were cohesive. The simple PSO model did not perform as well as the hybrid approach in terms of clustering. When there was a good silhouette coefficient, it could be observed that the summation always tended to be lower because the devices were more cohesive with the centroid.

In relation to the greatest distance that was found between a device and a gateway (Table 15), it was observed that in some cases the greatest distance was greater than the range of the technology. In the LP model, this distance was always less than the radius, while in the K-means model and the simple PSO and hybrid PSO models, values that were greater than the range were found, which occurred because these models did not adopt the range as a restriction to the clustering process. Out of the proposed models, the only method that guaranteed the range was the LP model; on the other hand, this model was the only one to present a negative silhouette coefficient, as shown in Table 9. In the LP model, the gateways were activated to serve the devices that were in range. So, the larger the range, the more likely the silhouette coefficient was to be bad. Although the clustering models did not use the range as a criterion, they clustered the devices by considering the best position; in this case, it was a clustering process, not a gateway activation process.

In the presented clustering models, the number of gateways was informed based on the results of the LP model. It was possible to enter a larger number of gateways and obtain the greatest distance within the range of the technology. It is worth noting that the idea was to quantify the smallest possible number of gateways and the model achieved the expected result, as did the clustering process.

Throughout Section 5.1–Section 5.3, the experiments were described considering the same parameters for each proposed model. It was concluded that the models corresponded to expectations. The experiments with the LP model only had one sample. In the performed tests, it was found that repetition with several samples and the same parameters returned the same result, matching the search for the best result. The clustering models presented different results for each sample because a random initial position was defined for each sample, which interfered with the final results. For evaluation purposes, 32 samples were run and the average of the silhouette coefficients and the total distances within the clusters were calculated to compare the models.

Table 16 presents the parameters that were applied in the experiments. In order to compare the results, six experiments were performed, all of which were applied in the INF scenario with an area of 2425 m${}^{2}$ in an indoor environment.

The results presented in Table 17 and Table 18 show a comparison between the six experiments, using the total distance and the silhouette coefficient as metrics. The higher the total distance, the greater the distance between the devices and their respective gateways. The silhouette coefficient represented the cohesion of the clusters.

When analyzing the results, the smallest distance found was from the hybrid PSO model in Experiment 1 and the largest distance was from the LP model in Experiment 6. In almost all of the experiments, the largest distance found was from the LP model.

When comparing the total distances in each experiment, it was found that the hybrid PSO model stood out as having the smallest distance in four experiments; the K-means model was second in this ranking. Regarding the greatest distance, the LP model achieved the greatest total distance in four experiments, followed in the ranking sequence by the simple and hybrid PSO models.

The same evaluation was conducted for the silhouette coefficient. The lowest silhouette coefficient out of the experiments was verified using the LP model in Experiment 6, which was equivalent to the highest distance summation that was found. As can be observed in Figure 6f, there was no cohesion between the devices and the gateways as the negative coefficient that was ascertained by the model characterized a high degree of dispersion. The K-means model showed the best silhouette coefficient in Experiment 5, with a value above 0.5; the closer to 1, the more cohesive the cluster. There were other results of above 0.5: Experiment 3 with the hybrid PSO model ranked second; Experiment 3 with the K-means model and Experiment 5 with the hybrid PSO model ranked in joint third position. When only comparing the models within the same experiment, it could be seen that the hybrid PSO model stood out in four experiments (1, 3, 4, and 6) and the K-means model stood out in two (2 and 5). The smallest silhouette coefficients were from the LP model, which achieved the smallest results in four experiments (3, 4, 5 and 6), followed by the simple PSO model with the smallest results in Experiments 1 and 2.

It was possible to observe that each model had its own contribution that could be improved by inserting other parameters or constraints. The K-means and hybrid PSO models were the best models for clustering, but the linear programming model was the only one that restricted the devices within a range.

A statistical analysis using the Friedman test is presented in Section 5.5.

### 5.5. The Friedman Test

The Friedman test is a non-parametric statistical test that was developed by Milton Friedman, who was an economist, statistician, and writer [35]. This test is used to detect differences between treatments in various experiments, allowing a choice to be made between two or more hypotheses using the data from a given experiment. The objective of this test is to determine whether there are at least two samples that represent the populations of distinct means out of a set of *n* samples (n≥2). In this way, it is possible to detect significant differences between the behaviors of two or more metaheuristics [36].

The Friedman test was applied to our experiments in order to evaluate the optimization models. The arithmetic means of the silhouette coefficients and the summation of the distances were used as metrics.

The Friedman test is based on ranking the data, so the lowest ranking value is assigned to the best performing algorithm. This test returned a *p*-value that allowed the similarities between the proposed models to be evaluated. The *p*-value was compared to an α value, which represented the significance of the test. α=0.05 was adopted; when the *p*-value <α, it was concluded that the algorithms were different. The degree of confidence was equal to 1−α. For α=0.05, we obtained a confidence level of 0.95 (or 95%).

Although the results showed whether or not there was a difference between the algorithms, it was not possible to know what was different. In view of this, the Friedman test compared the models and evaluated the differences between them. This comparison is called a post hoc test. Each comparison returned a value of *p*, which represented the similarity between the compared algorithms.

The tests were applied using the Keel (Knowledge Extraction based on Evolutionary Learning) software, which is a free software that was developed in Java by a group of researchers from Spain and the UK and is capable of performing various experiments involving data mining, including the Friedman test [36].

The test was applied using the data from the experiments presented in Table 17 and Table 18. The results are shown in Table 19, Table 20, Table 21 and Table 22. The standard deviations and the arithmetic means and medians of the silhouette coefficients were considered, along with the total distances between the gateways and the IoT devices.

When analyzing the ranking results presented in Table 19 and Table 21, it could be observed that the models all had a degree of similarity of less than 0.05, indicating that the optimization models were different. When analyzing the rankings, the hybrid PSO model performed the best in terms of the silhouette coefficient metric. In terms of the sum of the distances between the devices and the gateways, we obtained a different result for each metric.

When evaluating the post hoc tests, which aimed to compare the models to each other, it was observed that the K-means and hybrid PSO models had a *p*-value of greater than 0.05 in all of the presented cases, which confirmed that these metaheuristics were similar. It is noteworthy that the hybrid PSO model initialized the particles using the same initialization technique as the K-means model, which justified the similarity that was found in the Friedman test. The LP and simple PSO models showed a similarity when evaluating the arithmetic means of the silhouette coefficients and the sums of the distances between the gateways and the IoT devices.

The four models presented in this paper contributed to the planning and deployment of IoT gateways in a smart campus environment. Based on the results, it was possible to choose which model best fit a specific case. In Section 6, the final considerations of this work are presented.

## 6. Conclusions

This paper presented an approach for planning and deploying IoT gateways in smart campus environments, using the minimum number gateways that was required for the desired area coverage. The application scenario for the experiments was the Samambaia Campus of the UFG. In this environment, some departments were chosen for indoor and outdoor experiments. The heterogeneity of IoT devices led us to think about environments with multiple communication technologies, considering the characteristics of each technology. Thus, the proposed model focused on the LoRa, Wi-Fi, and BLE technologies.

The linear programming-based model considered the desired area, the range of the technology, the consumption capacity of the data link, and the deployed IoT devices to return the lowest possible number of gateways and to subsequently position them. For positioning, we presented the linear programming, K-means, and PSO models.

The experiments were applied to four scenarios (INF, academic blocks, Samambaia Campus, and IFTO Palmas) and achieved considerable results for both the quantification model and the gateway positioning models. The problems that were addressed by the model have been considered as difficult to solve and as the number of devices increased, the size of the area and the demand for processing and memory also increased.

With the results of these experiments, a comparative analysis was conducted that allowed for the evaluation of the behavior of each model and the determination of their advantages. It was concluded that the results were satisfactory and proved the efficiency of the models in relation to the proposed objective. The IoT gateway quantification was accurate and respected the established range and data demands. The positioning models defined the positions of the gateways and created clusters of devices that were associated with a gateway.

With the presented optimization models, it would be possible to plan the required area coverage for establishing wireless communication technologies. This planning would reveal the amount of communication equipment that is needed and would define the best positions for that equipment to be installed.

This work offers contributions to the field of IoT gateway planning and deployment through the optimization model. Throughout the research, we envisioned studies that could be developed in the future.

For future work, we intend to improve the optimization models, both from a quantification and positioning point of view by:

- Considering other parameters and constraints that may contribute to gateway quantification, with the goal of obtaining results that are even closer to the existing design;
- Associating weights with the objective functions or processes that could improve gateway positioning using the clustering models;
- Working on a model that can consider all three communication technologies simultaneously for gateway quantification.

## Figures and Tables

**Table 1:** The measurements of the areas that were used in the use case.

Desired Area	Size of Area (m^2^)	Type of Environment
INF	2.425	Indoor
Samambaia Campus	761.380	Outdoor
Academic Blocks	26.664	Indoor

**Table 2:** Comparison of network planning related works.

Author	Objective	Method of Optimization	Technology Comunication	Quantifies
Gravalos, Ilias, et al. [10]	Minimize coast/ QoS	LP	-	Yes
Ali, Hafiz Munsub, et al. [14]	Minimize coast	evolutionary algorithms	-	No
Maiti, Prasenjit, et al. [29]	Minimize latency	randomized, greedy, k-median, K-means and simulated annealing	-	No
Karthikeya, Surabhi Abhimithra, J. K. Vijeth, and C. Siva Ram Murthy [15]	Minimize coast	Heuristics	Wi-Fi, ZigBee, RFID	Yes
Matni, Nagib, et al. [30]	Minimize coast/ QoS	Fuzzy C-means	Lora	Yes
Lima, Marlon Paolo, Eduardo G. Carrano, and Ricardo HC Takahashi [16]	Minimize AP/ load	Genetic. Algorithm	Wi-Fi	Yes
Capone, Antonio, et al. [17]	Minimize coast/ Max. energy efficiency	LP	-	Yes
Wu, Wenjia, Junzhou Luo, and Ming Yang [18]	Minimize gateways/ position	LP/ Heuristics	-	No
Lin, Po-Chiang [19]	Minimize coast	LP	-	No

**Table 3:** The variables that were used for the LP optimization model, including the input parameters and data that were generated from the input parameters.

Variable	Description
*r*	Range of the communication technology
*n*	Number of deployed devices
*c*	Device bandwidth consumption
cb	Total link communication capacity
$A_{i,j}$	All possible points within the desired area
$D_{i,j}$	Coordinates of the deployed devices
$Mdist_{i,j}$	Distance matrix (measured between the point of a device and the points of the other deployed devices)
$Mativ_{i,j}$	Binary matrix (where 0 indicates that the distance between the devices is not within range and 1 indicates that the distance between the devices is within the range of the technology)

**Table 4:** The variables that were used in the K-means clustering model, including the input parameters.

Variable	Description
*k*	Number of gateways/devices to be deployed equals the number of centroids
*n*	Number of deployed devices
$D_{i,j}$	Coordinates of the deployed devices
$A_{i,j}$	All coordinates of the possible points within the desired area

**Table 5:** The variables that were used for the PSO model.

Variable	Description
*n*	Number of deployed devices
*g*	Number of gateways/centroids
$A_{i,j}$	Desired area/all possible points
$D_{i,j}$	Coordinates of the deployed devices
*w*	Inertia factor ($w_{initial}/w_{final}$)
mi	Maximum iterations
c1,c2	Constants (equivalent to the cognitive and social coefficients)
r1,r2	Random constants (generated with values between 0 and 1)
$pbest_{i}$	Best position of each particle
gbest	Best global position
$p_{i}$	Position vector of particle *i* within the search space
$v_{i}$	Velocity vector of particle *i*

**Table 6:** The ranges of the communication technologies that were adopted in the use case.

Technology	Range (m)	Environment	Reference
BLE	5	Indoor	[32]
Wi-Fi	25	Indoor	[33]
LoRa	70	Indoor	[34]

**Table 7:** The matrix of the distances between devices, which was used by the LP model to generate the activation matrix.

	0	1	2	3	4	5	6	7	8	9
0	0.0	25.5	10.2	10.44	21.38	11.7	11.4	21.93	23.77	22.36
1	25.5	0.0	18.38	33.42	7.28	13.89	34.06	20.22	5.0	3.16
2	10.2	18.38	0.0	15.26	12.53	6.08	15.81	12.21	15.26	15.62
3	10.44	33.42	15.26	0.0	27.78	19.8	1.0	23.02	30.53	30.48
4	21.38	7.28	12.53	27.78	0.0	10.0	28.3	13.04	2.83	6.08
5	11.7	13.89	6.08	19.8	10.0	0.0	20.52	15.81	12.17	10.82
6	11.4	34.06	15.81	1.0	28.3	20.52	0.0	23.09	31.06	31.14
7	21.93	20.22	12.21	23.02	13.04	15.81	23.09	0.0	15.3	19.0
8	23.77	5.0	15.26	30.53	2.83	12.17	31.06	15.3	0.0	5.0
9	22.36	3.16	15.62	30.48	6.08	10.82	31.14	19.0	5.0	0.0

**Table 8:** The matrix that was used by the LP model to generate the gateway activation.

	0	1	2	3	4	5	6	7	8	9
0	1	0	1	1	1	1	1	1	1	1
1	0	1	1	0	1	1	0	1	1	1
2	1	1	1	1	1	1	1	1	1	1
3	1	0	1	1	0	1	1	1	0	0
4	1	1	1	0	1	1	0	1	1	1
5	1	1	1	1	1	1	1	1	1	1
6	1	0	1	1	0	1	1	1	0	0
7	1	1	1	1	1	1	1	1	1	1
8	1	1	1	0	1	1	0	1	1	1
9	1	1	1	0	1	1	0	1	1	1

**Table 9:** The number of required gateways considering the INF area and bandwidth consumption variations.

Tech Comms	Quantity Devices	Range (m)	Gateways (Demand 3%)	Silhouette (Demand 3%)	Gateways (Demand 0%)	Silhouette (Demand 0%)
BLE	100	5	35	0.35794	35	0.35794
BLE	300	5	37	0.28183	37	0.29547
Wi-Fi	100	25	4	0.10635	3	0.35984
Wi-Fi	300	25	10	−0.07833	3	0.33021
LoRa	100	70	4	−0.23305	1	-
LoRa	300	70	10	−0.15928	1	-

**Table 10:** The result of the gateway quantification experiments in the academic blocks scenario with 300 devices that were communicating via Wi-Fi technology, as presented in Figure 7b.

Quantity of Devices/Gateways	Increased Distance Between Device/Gateway	Sum of the Distance Between Device/Gateway	Cluster	Silhouette Coefficient
21	25	338.62	0	0.31363
19	25	286.12	1	
21	24.7	361.36	2	
17	24.02	205.54	3	
16	24.7	263.59	4	
20	25	283.56	5	
10	22.56	153.04	6	
11	24.35	143.4	7	
17	24.08	231.6	8	
18	24.17	242.7	9	
11	24.76	179.06	10	
15	25	268.54	11	
13	24.33	185.21	12	
15	23.41	230.15	13	
13	23.19	197.4	14	
16	24.02	247.65	15	
10	23.71	155.17	16	
31	24.7	499.38	17	
6	22.47	107.08	18	
Total Distance:		4579.17		

**Table 11:** The results of the experiments with variations in the demand for data link consumption (LP method).

Area	Technology	Quantity of Devices	Demand	Quantity of Gateways	Silhouette Coefficient
INF	Wi-Fi	100	0	3	0.35984
INF	Wi-Fi	100	2	3	0.34473
INF	Wi-Fi	100	3	4	0.09411
INF	Wi-Fi	100	4	4	0.05009
INF	Wi-Fi	100	5	5	0.06368
INF	Wi-Fi	100	10	10	−0.12747
INF	Wi-Fi	100	15	17	−0.20703
INF	Wi-Fi	100	20	20	−0.21001
INF	Wi-Fi	100	30	34	−0.38046

**Table 12:** The silhouette coefficients after applying the K-means model, considering the number of gateways that resulted from the application of the LP model.

Communication Technology	Number of Devices	Range (m)	Number of Gateways (Demand 3%)	Silhouette Coefficient (Demand 3%)
BLE	100	5	35	0.42388
BLE	300	5	37	0.38236
Wi-Fi	100	25	4	0.50848
Wi-Fi	300	25	10	0.37390
LoRa	100	70	4	0.50848
LoRa	300	70	10	0.37390

**Table 13:** The silhouette coefficients after applying the PSO positioning model (simple approach), considering the number of gateways that resulted from the application of the LP model.

Communication Technology	Number of Devices	Range (m)	Number of Gateways (Demand 3%)	Silhouette Coefficient (Demand 3%)
BLE	100	5	35	0.23778
BLE	300	5	37	0.24597
Wi-Fi	100	25	4	0.45798
Wi-Fi	300	25	10	0.31159
LoRa	100	70	4	0.45150
LoRa	300	70	10	0.31159

**Table 14:** The silhouette coefficients after applying the hybrid PSO model, considering the number of gateways that resulted from the application of the LP model.

Communication Technology	Number of Devices	Range (m)	Number of Gateways (Demand 3%)	Silhouette Coefficient (Demand 3%)
BLE	100	5	35	0.36686
BLE	300	5	37	0.37331
Wi-Fi	100	25	4	0.50842
Wi-Fi	300	25	10	0.37530
LoRa	100	70	4	0.50842
LoRa	300	70	10	0.37530

**Table 15:** The distance summations and silhouette coefficients of the experiments that were performed in the three scenarios, considering 300 devices and Wi-Fi communication technology.

Model	Area	Range (m)	Number of Gateways	Summed Distance	Silhouette Coefficient	Increased Distance Between Devices and Gateways
LP	Samambaia Campus	100	31	17,359.73	0.38812	95.82
Simple PSO	Samambaia Campus	100	31	20,554.47	0.29233	120.49
Hybrid PSO	Samambaia Campus	100	31	14,796.27	0.40764	92.98
K-means	Samambaia Campus	100	31	14,965.47	0.40943	114.85
LP	Academic block	25	19	4604.6	0.30675	24.74
Simple PSO	Academic Blocks	25	19	5384.96	0.27088	24.52
Hybrid PSO	Academic Blocks	25	19	3982.1	0.39224	29.57
K-means	Academic Blocks	25	19	3974.86	0.38964	27.01

**Table 16:** The parameters that were applied in the experiments.

Experiment	Communication Technology	Range (m)	Number of Devices	Number of Gateways (Demand 3%)
1	BLE	5	100	35
2	BLE	5	300	37
3	Wi-Fi	25	100	4
4	Wi-Fi	25	300	10
5	LoRa	70	100	4
6	LoRa	70	300	10

**Table 17:** The comparison of the total distance metric (average), considering the experiments that were performed using the same parameters.

Experiment	K-Means Summed Distance	LP Summed Distance	Simple PSO Summed Distance	Hybrid PSO Summed Distance
1	196.80823	220.14000	315.47147	196.06500
2	809.21433	939.62000	1154.32925	1779.13501
3	885.48468	1289.35000	1056.12834	885.49252
4	1776.56160	4261.13000	2206.50263	1775.86463
5	885.50771	1289.35000	1050.06652	885.48468
6	1776.56160	8916.60000	2206.50263	1775.86463

**Table 18:** The comparison of the silhouette coefficient metric (average), considering the experiments that were performed using the same parameters.

Experiment	K-Means Silhouette Coefficient	LP Silhouette Coefficient	Simple PSO Silhouette Coefficient	Hybrid PSO Silhouette Coefficient
1	0.42334	0.35794	0.23778	0.42535
2	0.38212	0.28193	0.24597	0.37331
3	0.50842	0.09411	0.42984	0.50845
4	0.37390	−0.07833	0.31159	0.37530
5	0.50848	0.09411	0.45149	0.50842
6	0.37390	−0.15928	0.31159	0.37530

**Table 19:** The results from the Friedman test: the ranking applied to the arithmetic mean values and the sums of the distances between the gateways and the IoT devices.

Model	Ranking
LP	1.50000
Simple PSO	1.83330
K-means	3.33330
Hybrid PSO	3.33330
*p*-value	0.01694

**Table 20:** The results from the Friedman test: the comparison between each metaheuristic using the arithmetic means of the sums of the distances between the gateways and the IoT devices.

Comparison	*p*-Value With α = 0.05
K-means vs. LP	0.01391
LP vs. Hybrid PSO	0.01391
K-means vs. Simple PSO	0.04417
Simple PSO vs. Hybrid PSO	0.04417
LP vs. Simple PSO	0.65472
K-means vs. Hybrid PSO	1.00000

**Table 21:** The results from the Friedman test: the ranking applied to the arithmetic mean values of the silhouette coefficients.

Models	Ranking
Hybrid PSO	1.33330
K-means	1.66670
Simple PSO	3.33330
LP	3.66670
*p*-value	0.00200

**Table 22:** The results from the Friedman test: the comparison between each metaheuristic using the arithmetic means of the silhouette coefficients.

Comparison	*p*-Value With α = 0.05
LP vs. Hybrid PSO	0.00175
Simple PSO vs. Hybrid PSO	0.00729
K-means vs. LP	0.00729
K-means vs. Simple PSO	0.02535
K-means vs. Hybrid PSO	0.65472
LP vs. Simple PSO	0.65472

## Data Availability

Not applicable.
